# DeepEpiIL13: Deep Learning for Rapid and Accurate Prediction of IL-13-Inducing Epitopes Using Pretrained Language Models and Multiwindow Convolutional Neural Networks

**DOI:** 10.1021/acsomega.4c10960

**Published:** 2025-02-26

**Authors:** Cheng-Che Chuang, Yu-Chen Liu, Yu-Yen Ou

**Affiliations:** 1 Department of Computer Science and Engineering, Yuan Ze University, Chung-Li 32003, Taiwan; 2 Graduate Program in Biomedical Informatics, Yuan Ze University, Chung-Li 32003, Taiwan

## Abstract

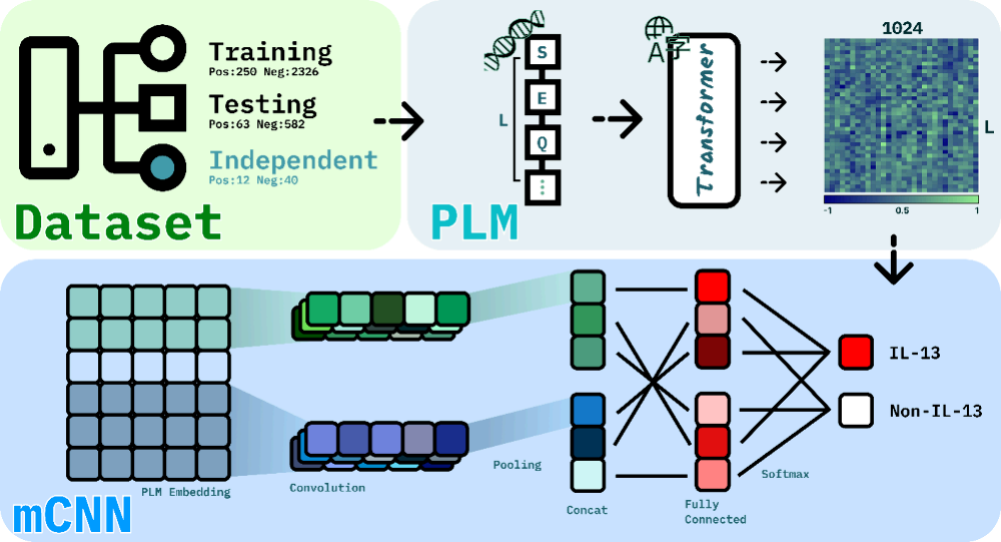

Accurate prediction of interleukin-13 (IL-13)-inducing epitopes is crucial for advancing targeted therapies against allergic inflammation, the cytokine storm associated with severe COVID-19, and related disorders. Current epitope prediction methods, however, often exhibit limitations in efficiency and accuracy. To address this, we introduce DeepEpilL13, a novel deep learning framework that uniquely synergizes pretrained language models with multiwindow convolutional neural networks (CNNs) for the rapid and accurate identification of IL-13-inducing epitopes from protein sequences. DeepEpilL13 leverages high-dimensional embeddings generated by the pretrained language model, which capture rich contextual information from protein sequences. These embeddings are then processed by a multiwindow CNN architecture, enabling the effective exploration of both local and global sequence patterns pertinent to IL-13 induction. The proposed DeepEpilL13 approach underwent rigorous evaluation using both benchmark data sets and an independent SARS-CoV-2 (severe acute respiratory syndrome coronavirus 2) data set. Results demonstrate that DeepEpilL13 achieves superior performance compared with traditional methods. On the benchmark data set, DeepEpilL13 attained a Matthews correlation coefficient (MCC) of 0.52 and an area under the receiver operating characteristic curve (AUC) of 0.86. Notably, when assessed on the independent SARS-CoV-2 data set, DeepEpilL13 exhibited remarkable robustness, achieving an MCC of 0.63 and an AUC of 0.92. These metrics underscore the enhanced predictive capability and robust applicability of DeepEpilL13, particularly within the context of the COVID-19 research and related viral infections. This study presents DeepEpilL13 as a powerful and efficient deep learning framework for accurate epitope prediction. By offering significant improvement in performance and robustness, DeepEpilL13 provides new and promising avenues for the development of epitope-based vaccines and immunotherapies specifically targeting IL-13-mediated disorders. The successful and rapid identification of IL-13-inducing epitopes using DeepEpilL13 paves the way for novel therapeutic interventions against a range of conditions, including allergic diseases, inflammatory conditions, and severe viral infections such as COVID-19, with potential for a significant impact on public health outcomes.

## Introduction

**Figure 1:**
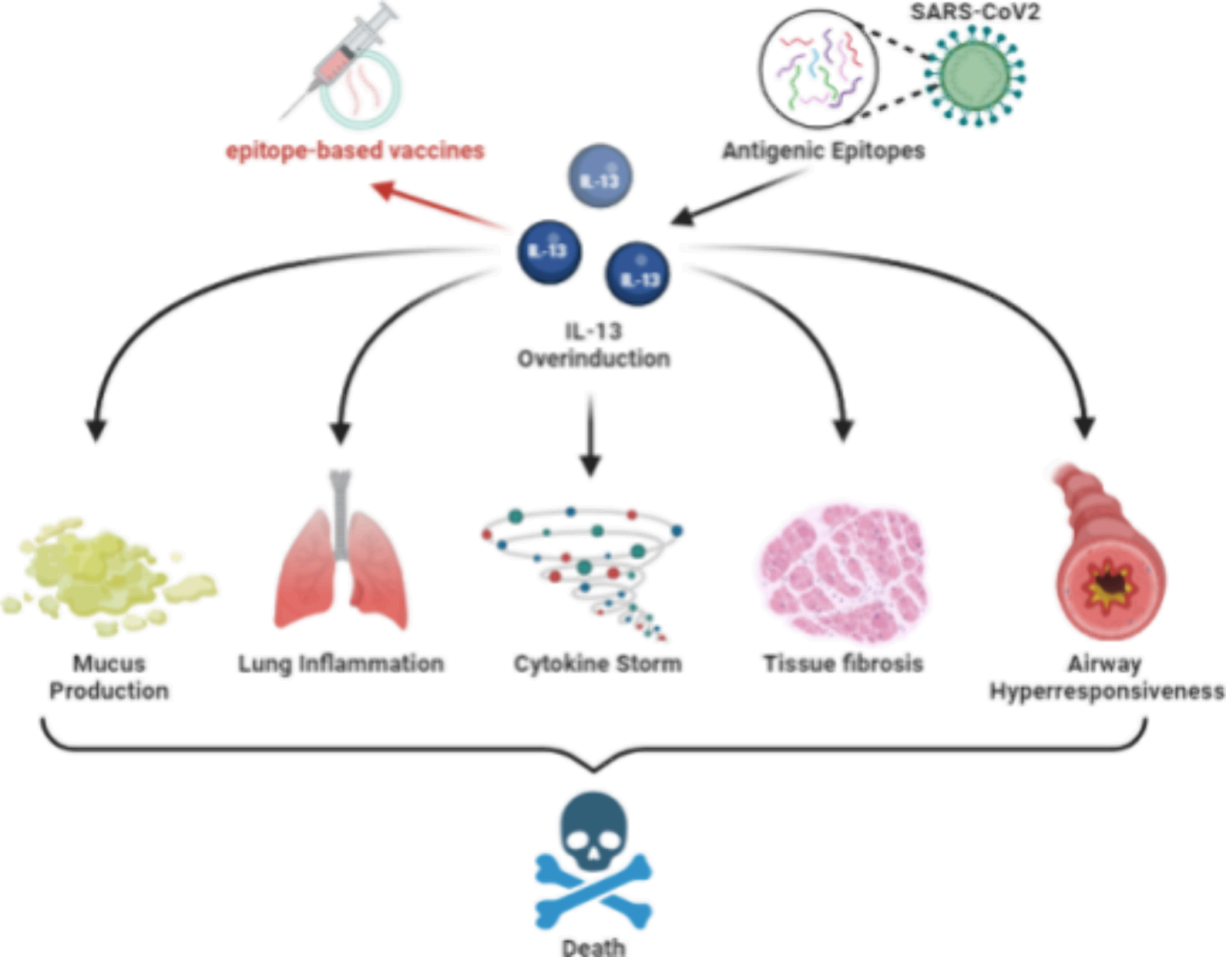
Schematic diagram of symptoms caused by overinduction of IL-13.

Interleukin-13 (IL-13) is a cytokine produced by Th2 cells, a subset of T helper cells, and plays crucial roles in the immune system.^1,2^ It stimulates B-cell proliferation and differentiation, enhances IgE antibody production, and contributes to various allergic diseases, such as allergic rhinitis and atopic dermatitis.^3,4^Figure 1 shows a schematic diagram illustrating the symptoms and pathological consequences caused by the overinduction of IL-13. The diagram depicts how excessive IL-13 production, often triggered by antigenic epitopes, can lead to a cascade of adverse effects including mucus production, lung inflammation, cytokine storm, tissue fibrosis, and airway hyperresponsiveness, ultimately contributing to conditions like severe allergic reactions and complications in viral infections such as COVID-19.^5^ This figure highlights the clinical relevance and therapeutic importance of targeting IL-13 and accurately predicting IL-13-inducing epitopes.

In the context of SARS-CoV-2 (severe acute respiratory syndrome coronavirus 2) infection, an immune overreaction known as a “cytokine storm” is particularly noteworthy.^6,7^ Characterized by the release of cytokines such as IL-13, this response not only fails to effectively contain the spread of the virus but may also lead to severe tissue damage and multiorgan failure.^8^ Recent medical research has confirmed that suppressing the overinduction of IL-13 can significantly reduce mortality and disease severity in mice infected with SARS-CoV-2.^9^ This discovery highlights the critical role of IL-13 in treating pulmonary diseases, especially playing a key role in the treatment of COVID-19.

One potential strategy involves developing epitope-based vaccines by incorporating viral antigenic epitopes that can induce IL-13 proliferation into immunogenic carriers.^10,11^ However, traditional methods for epitope identification, such as protein digestion, are often time-consuming and costly and may disrupt protein structures, affecting accurate epitope prediction.

In this study, we propose a deep learning approach to rapidly and accurately identify antigenic epitopes capable of inducing IL-13, with a focus on SARS-CoV-2. By leveraging pretrained language models like ProtTrans^12^ to extract protein sequence features and combining them with a Multiple Windows Scanning Convolutional Neural Network (mCNN) architecture, we aim to develop an efficient binary classifier for predicting IL-13-inducing epitopes.

This research has significant implications for developing novel vaccines and therapeutics targeting IL-13-related diseases including COVID-19. The proposed method promises to expedite the epitope identification process, thereby advancing immunotherapy development and potentially contributing to more effective prevention and treatment strategies against various allergic and inflammatory conditions.

## Materials and Methods

### Data Collection

In this study, two data sets were used; one was compiled by the developers of IL13Pred.^13^ To ensure a consistent basis for comparison, both the positive and negative data sets for this investigation were sourced directly from the original study. The data sets were then divided into a training set, which included 250 IL-13-inducing peptides and 2326 non-IL-13-inducing peptides, and a testing set, comprising 63 IL-13-inducing peptides and 582 non-IL-13-inducing peptides, as shown in Table1.

**Table 1:** Statistics of the Survey Dataset

data set	category	total	IL-13-inducing peptides	non-IL-13-inducing peptides
IEDB	training	2576	250	2326
	testing	645	63	582
SARS-CoV-2	independent	52	12	40

To further investigate whether antigenic epitopes of SARS-CoV-2 induce IL-13, the second data set was sourced from the IEDB,^14^ focusing on the study of the SARS-CoV-2 Spike glycoprotein. We identified 29 linear peptide epitopes as IL-13 inducing and 40 as non-IL-13 inducing within this context. To ensure the data set’s integrity, duplicates were removed, leading to a refined composition of 12 unique IL-13-inducing epitopes in the positive set, while the 40 non-IL-13-inducing epitopes were retained in the negative set.

### Sequence-Only Encoding

Sequence-only encoding converts the protein sequence of amino acids into an L* 20 binary matrix. For example, the amino acid “A” is encoded as a 20-dimensional binary vector [1,0,0,0,0,0,0,0,0,0,0,0,0,0,0,0,0,0,0,0,0,0,0,0,0,0,0,0,0], where the first position is 1, indicating the presence of “A”, and the remaining positions are 0s, representing the absence of other amino acids. This approach focuses on only whether amino acids are present or absent, excluding any additional data. It was employed as the initial set of features.

### Multiple-Sequence Alignment

Position-specific scoring matrices (PSSMs), derived from multiple-sequence alignments (MSAs) of homologous sequences, quantify the frequency of amino acid substitutions at each position while considering evolutionary relationships.^15^ These matrices provide a detailed representation (Lx20) of the likelihood of each amino acid occurrence throughout a protein’s sequence.

This technique enhances the accuracy of predictions for protein structure, interactions, and the identification of functional sites by incorporating evolutionary information.^16−18^ To compare different features with ProtTrans, we generated PSSMs using the MMseqs2^19^ algorithm on the SwissProt sequence database. These Lx20 matrices, corresponding to each l-residue protein, served as the second set of features.

### Pretrained Language Model Embeddings

ProtTrans^12^ utilizes pretrained Language Models (pLMs) to analyze a broad spectrum of known protein sequences, enabling the extraction of features that accurately represent sequence characteristics directly from the raw data. Compared to traditional sequence encoding methods, such as binary encoding or physicochemical property-based encoding, this approach offers richer biological insights, significantly improving the prediction accuracy for protein functions and interactions. Notably, several groups have advanced this field by proposing pretrained protein language models,^12,20−22^ among which ProtTrans stands out.

**Figure 2:**
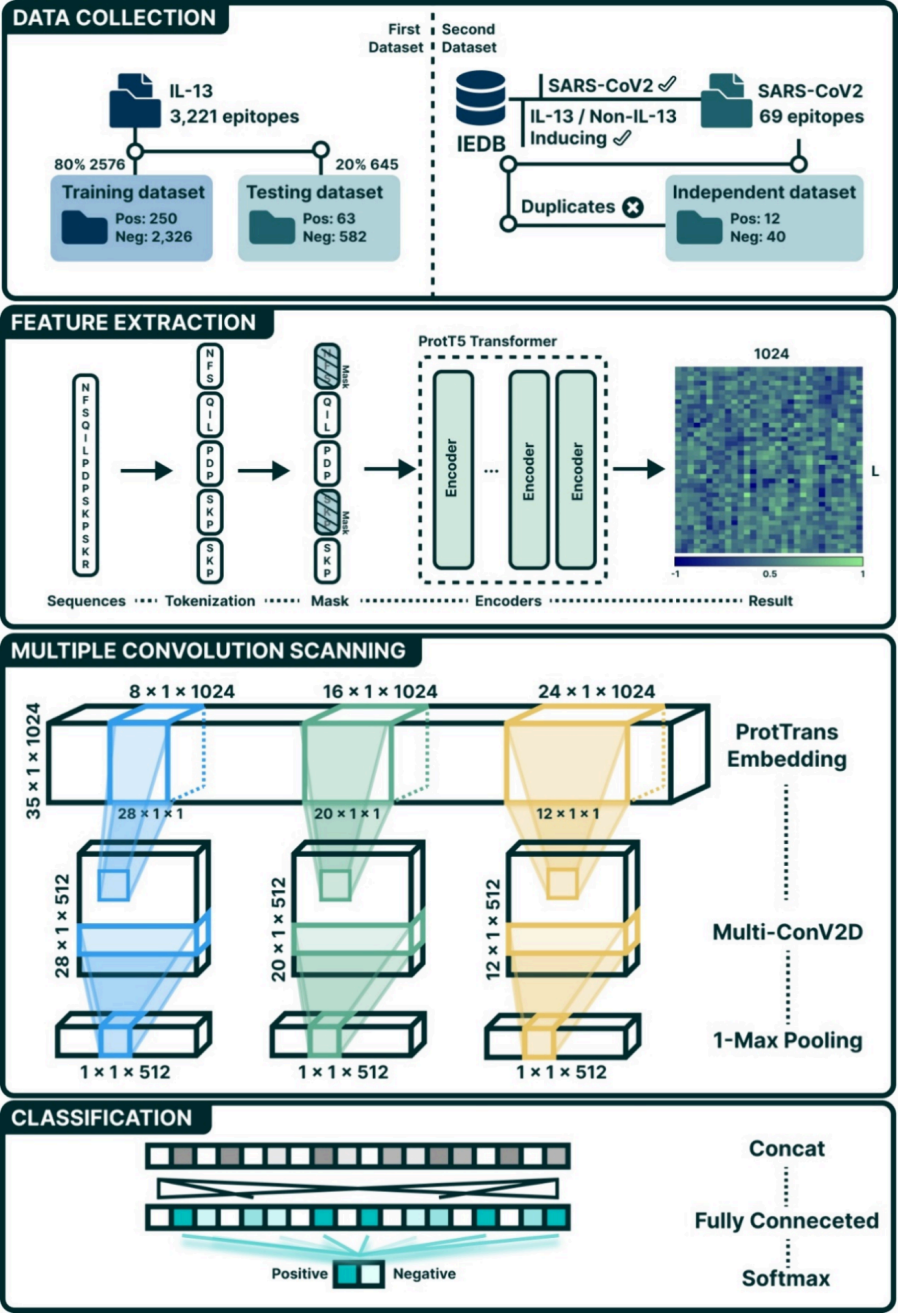
Workflow for the IL-13-inducing prediction model.

In our study, we employed pLMs, particularly the transformer-based ProtT5-XL-U50 (commonly referred to as ProtT5),^12,23^ to generate embeddings for our data sets, thereby enhancing our analytical capabilities. Using this method, embedding vectors of 1024 dimensions are derived from pretrained language models, encapsulating the contextual information on each amino acid. Furthermore, these vectors can be combined into a global vector that captures the entire protein’s essence. These vectors serve as inputs for the tasks of classifying IL-13-inducing epitopes. Figure 2 illustrates the workflow of the DeepEpilL13 model for IL-13-inducing epitope prediction. The workflow is divided into three main stages: Data Collection, Feature Extraction, and Classification. (1) Data Collection: Two data sets (IEDB benchmark and independent SARS-CoV-2) were used, showing the distribution of IL-13-inducing and noninducing epitopes. (2) Feature Extraction: Protein sequences are tokenized and fed into the ProtT5 Transformer model to generate 1024-dimensional embedding matrices. (3) Multiple Convolution Scanning: These embeddings are processed by a multiwindow Convolutional Neural Network (mCNN) with filters of varying sizes (8 × 1, 16 × 1, 24 × 1) followed by 1-Max Pooling and concatenation. (4) Classification: The concatenated feature vector is fed into a fully connected layer with a Softmax activation function for binary classification (IL-13 inducing vs noninducing).

### Multiple-Window Scanning Deep Learning Network Architecture

The multiwindow scanning approach, a technique initially developed for convolutional neural networks in natural language processing tasks such as sentence classification,^24^ has progressively proven its value in bioinformatics. In 2018, the DeepFam team^25^ recognized and pioneered its adaptation for protein sequence analysis. This foundational work was further advanced by models like MCNN-ETC^18^ in 2022, which successfully combined multiwindow scanning with evolutionary information derived from MSA. Building upon this established trajectory, our research in 2023 and ongoing work has focused on integrating multiwindow scanning with the powerful representations learned by pretrained protein language models (PLMs).^26−28^ This evolution reflects the increasing recognition of multiwindow scanning as a versatile and effective strategy for capturing complex patterns within biological sequences.

After obtaining different Lx1024 embedding matrices from ProtTrans^12^ for each sequence, to prevent the negative impact of varying sequence lengths in the data set on the classifier’s accuracy, we will standardize the data through an algorithm. This involves removing amino acid sequences longer than 35 residues and padding shorter ones with zeros to ensure all sequences are uniformly 35 residues long.

Next, within the multiwindow CNN model, each filter performs convolution with different fixed window sizes on these (35 × 1024) embedding features. This process aims to identify predictive local motifs that are indicative of IL-13-inducing epitopes based on the encoded contextual environment of each amino acid. After scanning various filters through a ProtTrans embedding matrix with a length of 35, each filter generates an output sequence of length *L* – *W* + 1, where *L* is the sequence length and *W* is the window size. For example, scanning with a single convolution filter of fixed window size 8 results in an output sequence with a length of length 28. The multiwindow model then expands this approach by employing multiple filters for each window size to capture a range of complementary features. By stacking filters with various window sizes, the model adeptly merges local and global sequence patterns. This strategy allows for the seamless integration of information from both the immediate and broader sequence contexts, enabling a more comprehensive analysis of sequence data.

Finally, the model applies a maximum pooling operation to the output of each filter, selecting the most significant feature value from each. The scalar outputs from these pooling operations, obtained from filters across various window sizes, are then concatenated into a single vector. This combined vector is fed into a fully connected layer to perform the final classification task of identifying IL-13-inducing epitopes.

### Performance Evaluation

To assess the model’s performance, various established metrics were employed, focusing on the five categories of predictions. These evaluation metrics included sensitivity, specificity, accuracy, the Matthews correlation coefficient (MCC), and the area under the receiver operating characteristic curve (AUC). These metrics were derived using formulas 1–4, described below:
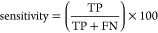
1
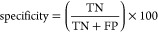
2
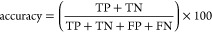
3
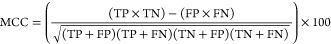
4

In this context, FP represents false positive, FN represents false negative, TP represents true positive, and TN represents true negative.

Finally, the area under the receiver operating characteristic curve (AUC) values spanning from 0 to 1, higher values denote superior model classification precision, whereas lower values imply subpar performance.

## Results and Discussion

The performance of the proposed multiwindow CNN model for classifying IL-13-inducing epitopes was rigorously evaluated using 5-fold cross validation on a benchmark training data set. This process facilitated fine-tuning the model to improve its metrics, including sensitivity, specificity, accuracy, the MCC, and AUC.

The model, specifically trained and tested on the benchmark data set comprising peptide sequences known to induce IL-13, underwent its final evaluation on an independent SARS-CoV-2 test set containing IL-13-inducing epitopes, ensuring its robustness and efficacy in identifying relevant peptides.

### Comparison Performance with Different Single-Window Sizes

Initially, CNN models were developed by integrating ProtTrans features, adhering to a single-window protocol, where the span of these windows extended from 8 to 34 residues, as shown in Table 2. A smaller window size of 8 achieved an MCC of 0.2352 and an AUC of 0.7249, while larger windows, such as 34 residues, had a higher MCC (0.3752) and AUC (0.8257). This suggests that larger window sizes are more effective at discerning broader patterns within the data but might also diminish the impact of detailed local interactions.

**Table 2:** Performance of IL-13-Inducing Epitope Classification with Varying Single Windows Using 5-Fold Cross Validation

window	sensitivity	specificity	accuracy	MCC	AUC
8	0.7090	0.6707	0.6743	0.2352	0.7249
16	0.7620	0.7127	0.7178	0.2995	0.7756
24	0.7613	0.7682	0.7675	0.3483	0.8251
32	0.7499	0.7628	0.7613	0.3376	0.8144
34	0.7019	0.8253	0.8133	0.3752	0.8257

### Comparison Performance with Different Window Combinations

Subsequently, a hierarchical approach was adopted to combine multiple window sizes, starting with a baseline of 34 residues and incrementally incorporating smaller windows, as detailed in Table 3. Adding windows of 24, in addition to the baseline of 34, resulted in an improved MCC of 0.3842 and an AUC of 0.8319. This illustrates the advantages of integrating local and global patterns through the use of a multiwindow architecture.

**Table 3:** Performance of IL-13-Inducing Epitope Classification with Varying Multiple Windows Using 5-Fold Cross Validation

window	sensitivity	specificity	accuracy	MCC	AUC
34	0.7019	0.8253	0.8133	0.3752	0.8257
34 24	0.7485	0.7991	0.7942	0.3842	0.8319
34 24 32	0.6867	0.8462	0.8311	0.4097	0.8316
34 24 32 16	0.7235	0.8196	0.8105	0.3932	0.8313
34 24 32 16 8	0.7414	0.8108	0.8047	0.4062	0.8309

### Comparison Performance with Different Filter Numbers

The performance of the model is also influenced by the number of filters applied to each window size. Table 4 illustrates how the model’s performance changes when the number of filters is adjusted between 64 and 1024. A minimal configuration of 64 filters resulted in an AUC of only 0.8229, whereas an excessively high count of 1024 filters led to a slight decrease in the AUC to 0.8242, possibly as a consequence of overfitting. The best results were achieved with a setting of 512 filters per window, which attained an AUC of 0.8319.

**Table 4:** Performance of IL-13-Inducing Epitope Classification with Varying Number of Filters Using 5-Fold Cross validation

filters	sensitivity	specificity	accuracy	MCC	AUC
64	0.7491	0.7833	0.7799	0.3573	0.8229
128	0.7226	0.8175	0.8086	0.3859	0.8269
256	0.6910	0.8407	0.8265	0.3994	0.8288
512	0.7485	0.7991	0.7942	0.3842	0.8319
1024	0.7266	0.8114	0.8036	0.3763	0.8242

### Comparison Performance with Different Features

**Figure 3:**
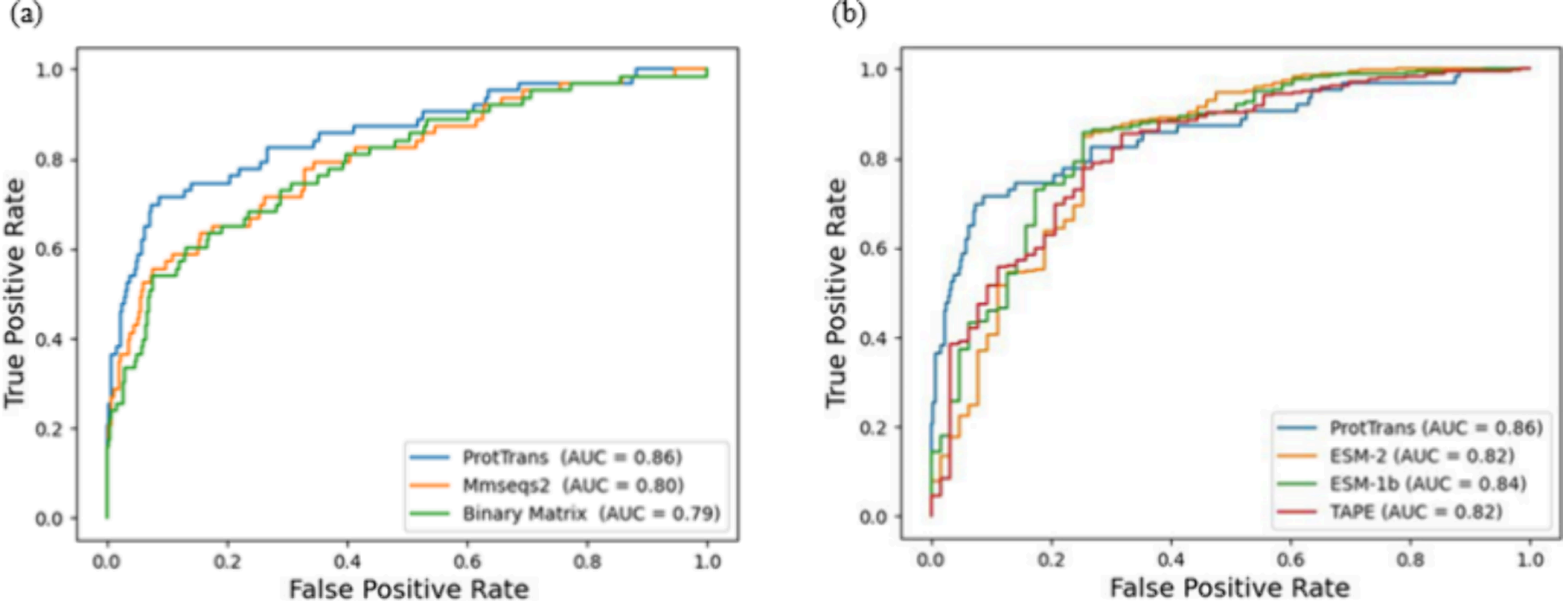
(a) ROC curve for the performance of predicting IL-13-inducing epitopes with different feature sets. (b) ROC curve for the performance of predicting IL-13-inducing epitopes with different protein language models.

The impact of ProtTrans embeddings on the multiwindow CNN model was assessed by experimenting with various input feature representations. Table 5 reveals that employing basic one-hot-encoded sequences led to a notably lower AUC of 0.7942. The addition of evolutionary information through position-specific scoring matrices (PSSMs) raised the AUC to 0.8015. Yet, ProtTrans features outperformed the others, securing the highest AUC of 0.8567, underscoring the effectiveness of leveraging contextual language models for feature representation. Figure 3a shows the receiver operating characteristic (ROC) curves comparing the performance of the multiwindow CNN model using different input feature representations: ProtTrans embeddings, MMseqs2-derived PSSMs (Mmseqs2), and Sequence-only Binary Matrix encoding. The ROC curves illustrate the trade-off between the True Positive Rate and False Positive Rate for each feature set. ProtTrans embeddings demonstrate the highest AUC (0.86), indicating superior performance in discriminating IL-13-inducing epitopes compared with PSSMs and binary matrix encoding.

**Table 5:** Prediction Performance of mCNN Models with Different Features

feature sets	sensitivity	specificity	accuracy	MCC	AUC
sequence-only encoding	0.6349	0.8299	0.8109	0.3356	0.7942
multiple-sequence alignment	0.6349	0.8436	0.8233	0.3531	0.8015
pretrained Language Model	0.7143	0.9124	0.8940	0.5227	0.8567

Based on the original ProtTrans study as well as relevant research findings, we selected ProtTrans, specifically the ProtT5-XL-U50 model, for its robust performance and advanced feature representation. As shown in Table 6, unlike earlier models, such as TAPE, which relied on shallower architectures, ProtT5-XL-U50 benefits from a transformer-based deep learning architecture that excels in capturing intricate sequence motifs and functional residues relevant to epitope prediction. Furthermore, when comparing the performance with other transformer-based protein models like ESM-2 and ESM-1b, ProtT5-XL-U50 consistently yielded higher AUC, accuracy, and MCC, demonstrating its effectiveness in this classification task. The results indicate that leveraging a pretrained model with deep contextual representations enhances peptide classification performance, making it an optimal choice for IL-13-inducing epitope identification. Figure 3b shows the ROC curves comparing the performance of various PLMs for predicting IL-13-inducing epitopes: ProtTrans, ESM-2, ESM-1b, and TAPE. The ROC curves show the trade-off between the True Positive Rate and False Positive Rate for each feature set. Among these, ProtTrans embeddings exhibit the highest AUC (0.86), indicating the best overall performance in discriminating epitopes. ESM-1b and ESM-2 also show competitive results, with AUCs of 0.84 and 0.82, respectively, while TAPE displays the lowest AUC of 0.82.

**Table 6:** Prediction Performance of mCNN Models with Different Protein Language Models

feature sets	sensitivity	specificity	accuracy	MCC	AUC
ProtTrans	0.7143	0.9124	0.8940	0.5227	0.8567
ESM-2	0.8488	0.7460	0.8388	0.4341	0.8246
ESM-1b	0.8591	0.7460	0.8481	0.4491	0.8393
Tape	0.8557	0.6825	0.8388	0.4018	0.8218

### Comparison Performance with Different Classifiers

**Figure 4:**
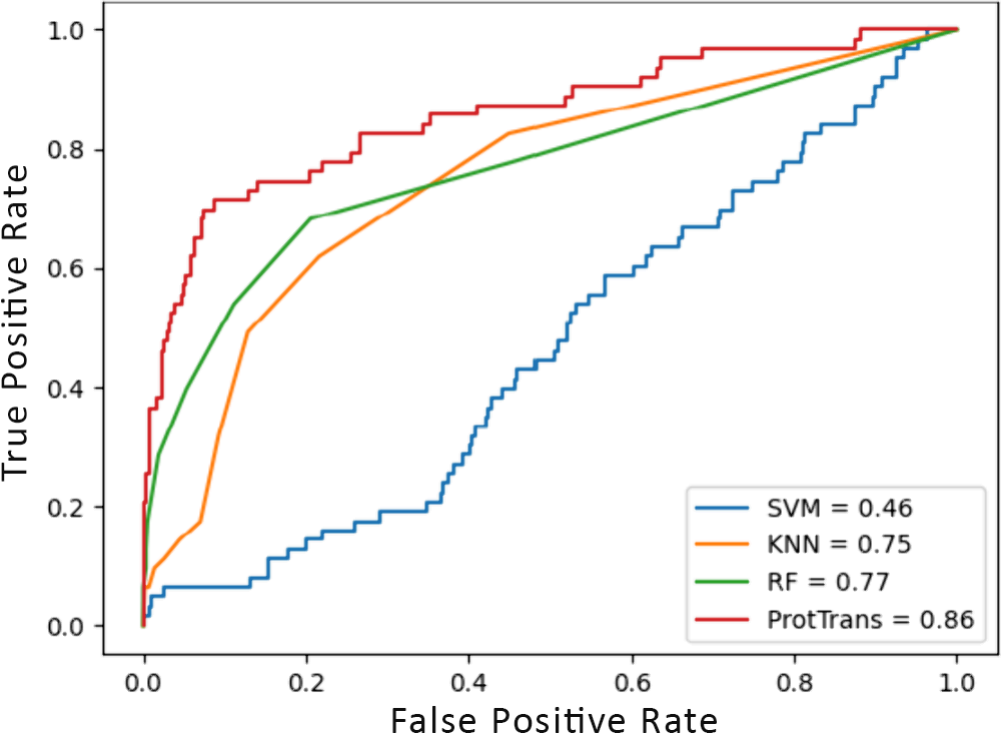
ROC curve for the performance of predicting IL-13-inducing epitopes with different classifiers.

Table 7 highlights the superior performance of the multiwindow ProtTrans-CNN model over traditional classifiers such as KNN, RF, and SVM, all utilizing ProtTrans profiles as their input. This innovative architecture achieved a significantly higher MCC of 0.5227 and an AUC of 0.8567 for classifying IL-13-inducing epitopes. In contrast, the best-performing conventional method, RF, achieved an MCC of only 0.3452 and an AUC of 0.7669. This notable improvement in performance underscores the advantages of combining ProtTrans embeddings with a multiwindow CNN architecture. By leveraging this approach, it becomes possible to more efficiently explore local and global patterns within sequence data, thus achieving better predictive outcomes in complex bioinformatics tasks. Figure 4 shows the receiver operating characteristic (ROC) curves comparing the performance of the multiwindow ProtTrans-CNN model with traditional machine learning classifiers (SVM, KNN, and RF), all utilizing ProtTrans embeddings as input features. The ROC curves demonstrate that the multiwindow CNN architecture (ProtTrans) significantly outperforms traditional classifiers, achieving a higher AUC (0.86) and demonstrating the effectiveness of the proposed deep learning approach for IL-13-inducing epitope prediction.

**Table 7:** Prediction Performance of Varying Classification Models with the Pretrained Language Model

classifier	sensitivity	specificity	accuracy	MCC	AUC
KNN	0.1429	0.9553	0.8760	0.1287	0.748
RF	0.1746	0.9948	0.9147	0.3452	0.7669
SVM	0.5873	0.4330	0.4481	0.0122	0.4640
mCNN	0.7143	0.9124	0.8940	0.5227	0.8567

### Comparison Performance with Previous Works

Table 8 compares the performance of the proposed model in classifying IL-13-inducing epitopes with that of previous techniques, including traditional machine learning models. The proposed model, utilizing ProtTrans features within a multiwindow CNN framework, achieved optimal results, marking a significant milestone with an MCC of 0.52 and an AUC of 0.86. This significantly exceeds the performance of earlier methods employing Pfeature,^30^ which recorded MCC of 0.3 and AUC of 0.8 in 2022, and MCC of 0.33 and AUC of 0.83 in 2023.

**Table 8:** Comparison with Existing Methods

method	year	sensitivity	specificity	accuracy	MCC	AUC
IL13Pred^a^	2022	0.718	0.73	0.729	0.3	0.8
iIL13Pred^a^	2023	0.746	0.757	0.7562	0.33	0.83
our method	2024	0.714	0.912	0.894	0.52	0.86

a These results are from iIL13Pred ref (29).

### The Prediction Performance with the SARS-CoV-2 Independent Data Set

The prediction performance of our model was further validated using an independent SARS-CoV-2 data set as shown in Table 9, where it achieved an impressive MCC of 0.63 and an AUC of 0.92. This outcome not only underscores the robustness and reliability of our model but also highlights its potential applicability in identifying key peptides in the context of current global health challenges. The significant performance on the SARS-CoV-2 data set reinforces the model’s efficacy in handling real-world data, paving the way for its contribution to the ongoing research and development in viral infection and vaccine design.^31,32^

**Table 9:** Prediction Performance of IL-13-Inducing Epitope Classification with the SARS-CoV-2 Independent Dataset

data set	sensitivity	specificity	accuracy	MCC	AUC
SARS-CoV-2	0.9167	0.8000	0.8269	0.6271	0.9167

### Extended Research: The Interleukins Coinduced by the Antigenic Epitopes that Induce Interleukin-13

In this study, we sought to delve deeper into the nature of the immune response elicited by IL-13-inducing epitopes, particularly in the context of SARS-CoV-2 and broader allergic and inflammatory conditions. Recognizing the complex interplay of cytokines in immune regulation, we extended our analysis beyond the primary induction of IL-13 to investigate the spectrum of interleukins coinduced by these antigenic epitopes. This exploration is critical, as the overall therapeutic outcome and potential side effects of epitope-based interventions are likely influenced not only by IL-13 induction but also by the broader cytokine milieu they trigger.

**Figure 5:**
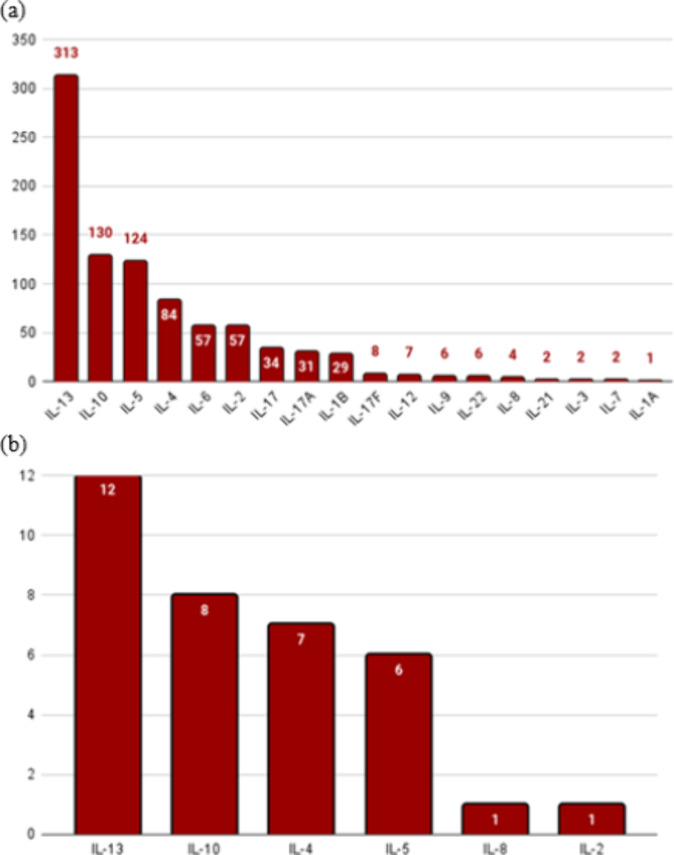
(a) Interleukins induced by the positive data set of the benchmark data set. (b) Interleukins induced by the positive data set of SARS-CoV-2.

Figure 5a presents a statistical overview of the interleukins coinduced by the positive data set within our benchmark data set. As expected, IL-13 is the most prominently induced interleukin. However, significantly, IL-10 and IL-5 are also notably coinduced, with substantial counts of 130 and 124 instances, respectively. Other interleukins such as IL-4, IL-6, and IL-2 are also induced, albeit to a lesser extent, indicating a more varied and nuanced immune response than that solely focused on IL-13. Analyzing the SARS-CoV-2 data set’s positive data set (Figure 5b) revealed a similar pattern of coinduction, although the specific counts differed. Importantly, despite data set variations, a consistent coinduction of IL-4, IL-10, and IL-5 alongside IL-13 is observed across both data sets.

The coinduction of IL-10 and IL-5 alongside IL-13 carries significant functional implications for the immune response and potential therapeutic applications. IL-10 is a well-established immunosuppressive cytokine with a potent regulatory functions. Its coinduction suggests a potential negative feedback loop or regulatory mechanism being activated concurrently with IL-13 induction. This could indicate an attempt to dampen or modulate the inflammatory response triggered by IL-13-inducing epitopes, potentially limiting excessive inflammation and tissue damage. Conversely, IL-5 is primarily known for its role in promoting eosinophil differentiation, activation, and survival, as well as B-cell growth and antibody production, particularly IgA. Coinduction of IL-5 could point toward a component of the response geared toward humoral immunity and eosinophilic inflammation, which is relevant in allergic diseases and some viral infections.

The observed coinduction profile, therefore, suggests that IL-13-inducing epitopes are not simply driving a unidirectional pro-inflammatory IL-13 response. Instead, they appear to trigger a more complex and multifaceted immune response involving a balance of pro-inflammatory (IL-13, IL-5, potentially IL-4, IL-6) and regulatory (IL-10) cytokines. Understanding this cytokine interplay is crucial for the rational design of epitope-based vaccines and immunotherapies. For instance, in the context of vaccine development, epitopes that preferentially induce IL-13 but minimize the coinduction of pro-inflammatory cytokines like IL-6, while enhancing regulatory cytokines like IL-10, might be desirable to achieve therapeutic efficacy with reduced immunopathology. Alternatively, for certain immunotherapeutic applications, it might be advantageous to select epitopes that promote a specific cytokine profile, perhaps favoring IL-13 induction while also leveraging the regulatory effects of coinduced IL-10 to fine-tune the immune response and avoid excessive inflammation. Further research is needed to fully elucidate the functional consequences of this coinduction pattern and to explore strategies to manipulate the cytokine profile for optimized therapeutic outcomes in IL-13-mediated diseases.

## Conclusions

In summary, this study introduces DeepEpilL13, a deep learning framework designed for the prediction of IL-13-inducing epitopes, integrating pretrained language models with mCNNs. A key aspect of DeepEpilL13 is its architecture, which aims to leverage the contextual embeddings from ProtTrans and the multiscale feature extraction of mCNNs to potentially address limitations of existing epitope prediction methods. This combination represents an incremental step in methodological development within the field of immunoinformatics.

The predictive performance of DeepEpilL13 was evaluated, and the results indicate a promising level of efficacy. On benchmark data sets, the model achieved an MCC of 0.52 and an AUC of 0.86, suggesting an improvement compared to previous approaches. Furthermore, validation using an independent SARS-CoV-2 data set demonstrated the model’s robustness and potential for practical application, with an MCC of 0.63 and an AUC of 0.92. These findings suggest that DeepEpilL13 could be a useful tool for identifying IL-13-inducing epitopes and exhibits potential for handling varied data sets relevant to real-world health challenges.

In conclusion, DeepEpilL13 may serve as a valuable bioinformatics resource to support the development of epitope-based vaccines and immunotherapies. Beyond its predictive capabilities, the framework may contribute to a better understanding of sequence patterns associated with IL-13 induction and the immune responses involving coinduced interleukins. By facilitating more precise epitope identification, DeepEpilL13 offers a potential avenue to accelerate the exploration and development of novel therapeutic interventions for IL-13-mediated disorders, including allergic diseases, inflammatory conditions, and viral infections such as COVID-19, with the prospect of positively impacting public health.

### Key Points

1. Develops a novel deep learning architecture combining pretrained protein language models and multiwindow convolutional neural networks for IL-13-inducing epitope classification.
2. Demonstrates the utility of large-scale pretrained language models like ProtTrans for enriching sequence representations with meaningful context.
3. Shows the benefits of multiwindow CNN architectures for capturing binding motifs at both local and global scales by scanning across diverse residue window sizes.
4. Makes code and data publicly available to facilitate further research building on pretrained protein encoders and multiwindow CNNs for IL-13-inducing epitope modeling.
5. It provides an impactful proof of concept for integrating representation learning and deep learning in protein sequence analysis.

## Data Availability

The code and data for this work are available at https://github.com/s1101404/DeepEpiIL13.
